# Consensus and evidence-based medication review to optimize and potentially reduce psychotropic drug prescription in institutionalized dementia patients

**DOI:** 10.1186/s12877-018-1015-9

**Published:** 2019-01-08

**Authors:** Mireia Massot Mesquida, Montserrat Tristany Casas, Alicia Franzi Sisó, Isabel García Muñoz, Óscar Hernández Vian, Pere Torán Monserrat

**Affiliations:** 1 Servei d’Atenció Primària Vallès Occidental, Institut Català de la Salut, Rambla Sabadell 229, 08201 Sabadell Barcelona, Spain; 2 Equip d’Atención Primària Arenys de Mar, Institut Català de la Salut, Arenys de Mar Barcelona, Spain; 3 Unitat de Suport a la Recerca Metropolitana Nord, Fundació Institut Universitari per a la recerca a l’Atenció Primària de Salut Jordi Gol i Gurina (IDIAPJGol), Mataró, Spain

**Keywords:** Medication review, Dementia, Psychotropic drugs, Nursing homes, Institutionalized patients

## Abstract

**Background:**

Dementia patients often show neuropsychiatric symptoms, known as behavioral and psychological symptoms of dementia (BPSD). These are a common motive for medical consultations, hospitalizations, and nursing home stays. Various studies have suggested that the high prevalence of psychotropic drug use to treat BPSD in institutionalized dementia patients may lead to impaired cognitive capacity, rigidity, somnolence, and other complications during the course of the illness. The aim of this study was to design a consensus-based intervention between care levels to optimize and potentially reduce prescription of psychotropic drugs in institutionalized patients with dementia and assess the changes occurring following its implementation.

**Methods:**

**Design**: Prospective, quasi-experimental, pre/post intervention, multicenter study. **Scope**: 7 nursing homes associated with a single primary care team. **Inclusion Criteria**: Institutionalized patients diagnosed with dementia and under treatment with 1 or more psychotropic drugs for at least 3 months. **Sample**: 240 individuals; mean age, 87 years (SD: 6.795); 75% (180) women. **Intervention**: Creation of evidence-based therapeutic guidelines for psychotropic drug use in the treatment of BPSD by consensus between reference professionals. Joint review (primary care and geriatric care nursing home professionals) of the medication based on the guidelines and focusing on individual patient needs. **Primary variable**: Number of psychotropic drugs used per patient. **Assessment**: Preintervention, immediate postintervention, and at 1 and 6 months.

**Results:**

Overall, the number of psychotropic drugs prescribed was reduced by 28% (from 636 before to 458 after the intervention). The mean number of psychotropic drugs prescribed per patient decreased from 2.71 at baseline to 1.95 at 1 month postintervention and 2.01 at 6 months (*p* < 0.001 for both time points). Antipsychotics were the drug class showing the highest reduction rate (49.66%). Reintroduction of discontinued psychotropic drugs was 2% at 1 month following the intervention and 12% at 6 months.

**Conclusions:**

A consensus guidelines-based therapeutic intervention with a patient-centered medication review by a multidisciplinary team led to a reduction in prescription of psychotropic drugs in institutionalized dementia patients.

**Electronic supplementary material:**

The online version of this article (10.1186/s12877-018-1015-9) contains supplementary material, which is available to authorized users.

## Background

Patients with dementia often show neuropsychiatric symptoms. These are known as behavioral and psychological symptoms of dementia (BPSD) and are a common motive for medical consultations, hospital admissions, and nursing home stays [1]. It is estimated that at least 90% of dementia patients will develop some type of BPSD over a period of 5 years, and this may have significant clinical implications in 85% of cases [2–4].

It is known that psychotic symptoms are more frequent during the moderate and severe stages of dementia, but the relationship between depression and dementia is not as clear. Some studies have suggested that there may be an inverse relationship, with depression present in the initial stages of the illness and absent in later ones. However, this notion may result from the difficulty in diagnosing depression in advanced stages of dementia, and not from an actual reduction in its prevalence [4]. Study of the natural history of dementia has shown that cognitive deterioration occurs more rapidly in patients with psychotic symptoms [5]. When these are treated with medication, periodic review of the treatment may be necessary [6], as the patient’s clinical status may change quickly, and along with it, the associated BPSD.

Psychotropic drug use in patients with dementia is currently quite high [7, 8]. An estimated 70% of dementia patients take 1 or more of these drugs [8–10]. In individuals over the age of 65, the prevalence of benzodiazepine consumption is 20% [11] and prescription of antidepressant agents is 40% [3]. In institutionalized dementia patients older than 65 years, benzodiazepine prescription reaches 47.1% and antidepressants 54%, whereas the use of antipsychotic drugs is estimated at 24.8 to 39.9% [8, 12].

Extensive use of psychotropic drugs to treat BPSD in institutionalized patients may lead to decreased cognitive capacity, rigidity, or somnolence, and result in complications such as pneumonia [13]. The EARCAS study [14], designed to explore the magnitude and implications of incidents related to patient safety in Spanish nursing homes, concluded that drug-related adverse events may be linked to a lack of medication review, with falls being the main adverse event. In addition, 96.6% of the experts consulted stated that the patients at the highest risk of experiencing a drug-related adverse event are those with impaired cognitive function (eg, Alzheimer disease, coma, dementia), and particularly those with behavioral disorders. In parallel, Billioti et al. [11] found that benzodiazepine use is associated with an increased risk (around 50%) of experiencing dementia. In a cohort study including 60,746 patients, Coupland et al. [15] examined the association between antidepressant use and serious drug-related adverse events in patients older than 65 years with depression. The authors reported a significantly higher rate of all-cause mortality in the group treated with antidepressants than in those who did not receive these drugs.

Several studies [16, 17] have cautioned about the risks of psychotropic drug use for treating BPSD and the need to periodically review the therapeutic strategy applied over time. This concern suggests that there may be a need for interventions aimed at reducing the risk associated with these drugs. The recent publication of several clinical trial protocols [18–21] intended to decrease the use of these drugs in institutionalized dementia patients further illustrates the growing interest in this issue.

Considering this background, the aim of this study was to design an intervention by consensus between specialized caregivers implicated in the management of patients with dementia (eg, neurologists, geriatric specialists) and primary care general practitioners (GPs) for optimizing and potentially reducing the prescription of psychotropic drugs in this population, and evaluate the changes occurring after its implementation.

## Methods

This is a prospective, multicenter, quasi-experimental, longitudinal, pre/postintervention study, conducted between 2012 and 2014. The patients included were residing in 7 nursing homes linked to a single primary care center in a semi-urban setting; the total population was 606 institutionalized patients, 40% with dementia. Drug prescription from the publically-funded national health system was carried out by the GPs in the primary care center. Dementia patients in these homes receiving pharmacological treatment with 1 or more psychotropic drugs from the Anatomical Therapeutic Chemical Classification System of the WHO [22] (ATC code N, with the exception of N01 anesthetics, N02 analgesics, and N07 others) for 3 months or longer were included. Patients with an active diagnosis of severe mental disorder (eg, personality disorder, severe depression, psychosis, schizophrenia) were excluded from the study. At 6 months following the intervention, all patients were assessed to determine whether some of the drugs that were discontinued during the intervention had been restarted or new drugs prescribed.

The main study variable was the number of psychotropic drugs prescribed for each patient before the intervention, immediately after the intervention, and at 1 month and 6 months of follow up. Secondary variables included the patients’ socio-demographic characteristics (age and sex) and drug-related variables (active ingredient, dose, and frequency of administration of all psychotropic drugs prescribed).

The intervention consisted of a review of the medications received by the participating patients, accomplished by individual, personal meetings between 1 primary care GP and 1 pharmacist, and the nursing home physicians and nurses. The intervention was developed as follows:

1. Creation of BPSD guidelines by consensus (Additional file 1). A multidisciplinary group of referral professionals was formed, consisting of a neurologist, a psychiatrist, a geriatrician, 2 GPs and 4 pharmacists, who designed the therapeutic guidelines for treating BPSD. The guidelines were evidence-based and included individual recommendations for pharmacological and nonpharmacological management of the behavioral and psychological symptoms, grouped as follows: apathy, aggression, anxiety, agitation, depression, psychosis, and insomnia. A brief summary of the general content of the guidelines and the method used in the medication review is provided in Additional file 2.
2. Prior to implementation of the guidelines-based medication review, 1 GP and 1 primary care pharmacist underwent a training phase focused on management of patients with BPSD (eg, assessment of dementia patients, management of the medication implicated in their treatment, the criteria and scheduling for drug withdrawal, strategies for symptoms that did not respond to treatment). Both these professionals had participated in designing the guidelines and were in charge of carrying out the fieldwork in the nursing homes. The training was carried out in person, was led by a neurologist, geriatrician, and psychogeriatrician, and was based on the contents of the guidelines. In addition to discussion about the theoretical framework, several case examples were raised and examined. A practical training session was then carried out in institutionalized dementia patients prescribed psychotropic drugs, where the trainees applied what had been discussed during the training before the start of the project.
3. Review of the treatment plan: A timeline for nursing home visits was created to review the course of the treatment. Before each scheduled visit (work session) with the nursing home physician and nurse, the medication patients were receiving underwent a preliminary evaluation by the GP and pharmacist to detect any incidents related to the prescription (eg, duplicates, inappropriate drugs) and to determine which prescription-related aspects required evaluation of patients for decision-making (continue or discontinue a drug).
4. Work session in the nursing home: Before conducting the joint medication review, the primary care GP and pharmacist asked the nursing home physician and nurse to confirm the patients’ dementia diagnosis to include them in the study. These professionals then established the patients’ current status with regard to their prognosis, level of dependence, and frailty to facilitate decisions on the need to adapt the intensity of treatment, change treatment, or discontinue treatment based on the benefit-risk for the patient [23]. The assessment of the patient’s current status included the severity of dementia (Global Dementia Scale [GDS]), level of dependence (Barthel index score), cognitive state (Pfeiffer test), and prognosis (in end-of-life patients). Treatment assessment included the indication, effectiveness, and safety (eg, duplication, interactions, contraindications) of the drugs prescribed. Based on these joint evaluations, changes in the patient’s treatment plan considered to be appropriate and relevant were proposed.
5. Follow-up: Follow-up of the consensus changes was carried out at 1 and 6 months to quantify the reintroduction of psychotropic drugs that had been discontinued during the work sessions, changes in the dose of psychotropic drugs that continued in prescription, and prescription of any new psychotropic drug.

Patient-related data were provided by the nursing home professionals and prescription data were obtained from the patients’ medical records. The information was entered in an anonymous database by one of the authors (MTC) at each of the study time points. The data collected were reviewed to ensure accurate recording.

### Statistical analysis

In the univariate analysis, quantitative variables are expressed as the mean and standard deviation (SD) and qualitative variables as the percentage. The Student-t test for paired data was used to compare the mean number of psychotropic drugs prescribed before (baseline) and after the intervention and between baseline and 1 and 6 months. The Wilcoxon test was used to analyze this variable in each nursing home. The sample size was not calculated a priori, as all patients meeting the inclusion criteria were enrolled. The G*power program [24] was used to analyze the statistical power of the study a posteriori, to determine whether there was a loss of power due to the sample size.

## Results

**Fig. 1:**
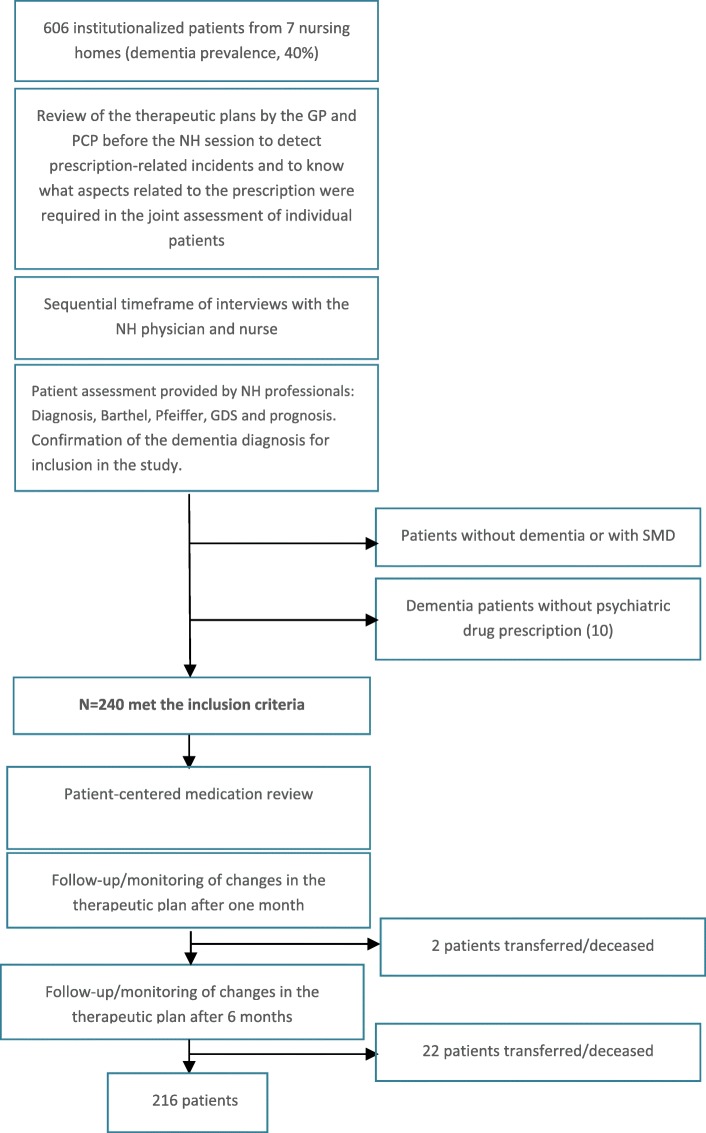
Flow chart of patient inclusion at each point of the intervention. Abbreviations: *GDS* Global Dementia Scale, *GP* general practitioner, *NH* nursing home, *PCP* primary care pharmacist, *SMD* severe mental disorder

From a total population of 606 institutionalized individuals, 250 patients had a diagnosis of dementia; 240 of the 250 (96%) were receiving 1 or more psychotropic drugs for at least 3 months and were included in the study Over follow-up, 2 patients (1.67%) were lost at 1 month and 22 patients (10%) were lost at 6 months due to death or a change of nursing home (Fig. 1).

Mean patient age was 87.09 years (SD: 6.795), and 75% (*n* = 180) were women. There were no significant differences in patient age between the participating nursing homes. The characteristics of the study population in each nursing home are shown in Table 1.

**Table 1:** Patient age and sex, and percentage of patients with dementia in each nursing home

Nursing home	Patients with dementia and PD, n (%)	Prevalence of dementia, %	Mean age, y (SD)	Women, n (%)
NH 1	35 (14.6%)	35.0%	87.98 (7.78)	28 (80%)
NH 2	42 (17.5%)	43.8%	88.05 (4.99)	38 (90.5%)
NH 3	25 (10.4%)	24.0%	87.44 (6.25)	15 (60%)
NH 4	14 (5.8%)	35.0%	89.57 (8.22)	10 (71.4%)
NH 5	52 (21.7%)	45.2%	85.25 (7.51)	32 (61.5%)
NH 6	50 (20.8%)	58.1%	86.66 (7.35)	37 (74%)
NH 7	22 (9.2%)	33.9%	87.36 (3.63)	20 (90.9%)
TOTAL	240	39.6%	87.9 (6.8)	180 (75%)

*NH* nursing home, *PD* psychotropic drugs, *SD* standard deviation, *y* years. Patients with dementia: patients evaluated by a neurologist with a confirmed diagnosis of dementia according to ICD-10 criteria

In total, 636 psychotropic drugs were prescribed before the intervention and 458 after; this latter value includes 216 drugs that were deprescribed and 38 new drugs that were prescribed; therefore, the final balance was 178 psychotropic drugs discontinued (28% decrease). The decrease was statistically significant (*p* < 0.0001) at all the time points analyzed relative to baseline. The pre- and postintervention difference in the number of drugs prescribed per patient was 0.755 (95% CI: 0.624–0.886); that is, 0.771 (95% CI: 0.635–0.908) at 1 month of follow-up and 0.634 (95% CI, 0.474–0.794) at 6 months (*p* = 0.000 in all cases). The medication changes occurring in relation to the intervention, including dosing changes, are summarized in Table 2. The decrease in number of psychotropic drugs per patient was statistically significant in all the participating nursing homes except one (Table 3). At 1 month after discontinuation, only 4 (2%) psychotropic drugs that had been discontinued were reintroduced, and at 6 months, 12% of drugs were restarted (11 antidepressants, 8 antipsychotics, and 3 hypnotics).

**Table 2:** Overall prescription-related changes following the intervention, and at 1 and 6 months

Drug prescription changes	Baseline	Postintervention	1 month	6 months
Dose increase, n		25	2	9
Dose decrease, n		34	11	6
Discontinued, n		216	15	43
Newly prescribed drugs, n		38	9	42
Reintroduced drugs, n		0	4	22
Patients 0 PD, n (%)	10 (4.00%)	32 (12.80%)	33 (13.31%)	25 (11.06%)
Patients 1–2 PD, n (%)	122 (48.80%)	151 (60.40%)	144 (58.06%)	134 (59.29%)
Patients 3–4 PD, n (%)	89 (35.60%)	60 (24.00%)	65 (26.21%)	55 (24.34%)
Patients > 4 PD, n (%)	29 (11.60%)	7 (2.80%)	6 (2.42%)	12 (5.31%)
Number psychotropic drugs/patient, mean (SD)	2.71 (1.47)	1.95 (1.24)	1.95 (1.26)	2.06 (1.36)
Difference (95% CI)	0.755 (0.624–0.886)* *p* = 0.000		0.771 (0.635–0.908)* *p* = 0.000	0.634 (0.474–0.794)* *p* = 0.000

Prescription-related changes and percentages of patients receiving psychotropic agents grouped into 4 categories (0, 1–2, 3–4, and > 4 psychotropic drugs) at the different time points. The calculation of percentages in each of the 4 categories was made over the total of patients with dementia in the nursing homes (*n* = 250). Difference in the mean number of psychotropic drugs prescribed before (baseline) and after the intervention and between baseline and 1 and 6 months are shown.

*Difference in the mean number of psychotropic drugs prescribed before (baseline) and after the intervention and between baseline and 1 and 6 months

**Table 3:** Number of psychotropic drugs per patient at the different assessment points in each nursing home

	Number psychotropic drugs/patient			
Nursing home (NH)	Baseline, mean (SD)	Post-intervention, mean (SD)	1 month, mean (SD)	6 months, mean (SD)
NH 1	2.71 (1.53)	2.06 (1.33)	2.06 (1.33)	2.32 (1.66)
	0.65 (*p* = 0.001)		0.65 (*p* = 0.001)*	0.39 (*p* = 0.035)*
NH 2	2.64 (1.30)	1.83 (1.14)	1.93 (1.21)	2.12 (1.35)
	0.81 (*p* = 0.000)		0.71 (*p* = 0.000)*	0.52 (*p* = 0.002)*
NH 3	3 (1.53)	2.24 (1.42)	2.24 (1.36)	1.95 (1.40)
	0.76 (*p* = 0.000)		0.76 (*p* = 0.001)*	1.05 (*p* = 0.000)*
NH 4	3.43 (1.87)	2.43 (1.65)	2.14 (1.70)	2.54 (1.94)
	1.00 (*p* = 0.004)		1.29 (*p* = 0.004)*	0.89 (*p* = 0.008)*
NH 5	3.1 (1.49)	1.94 (1.13)	1.98 (1.15)	2.04 (1.17)
	1.16 (*p* = 0.000)		1.12 (*p* = 0.000)*	1.06 (*p* = 0.000)*
NH 6	2.23 (1.26)	1.62 (1.04)	1.55 (1.14)	1.64 (0.98)
	0.61 (*p* = 0.000)		0.68 (*p* = 0.000)*	0.59 (*p* = 0.000)*
NH 7	2.23 (1.45)	2.14 (1.32)	2.14 (1.32)	2.23 (1.54)
	0.09 (*p* = 0.593)		0.09 (*p* = 0.593)*	0.00 (*p* = 1.000)*

*Difference in the mean number of psychotropic drugs prescribed before (baseline) and after the intervention and between baseline and 1 and 6 months

**Fig. 2:**
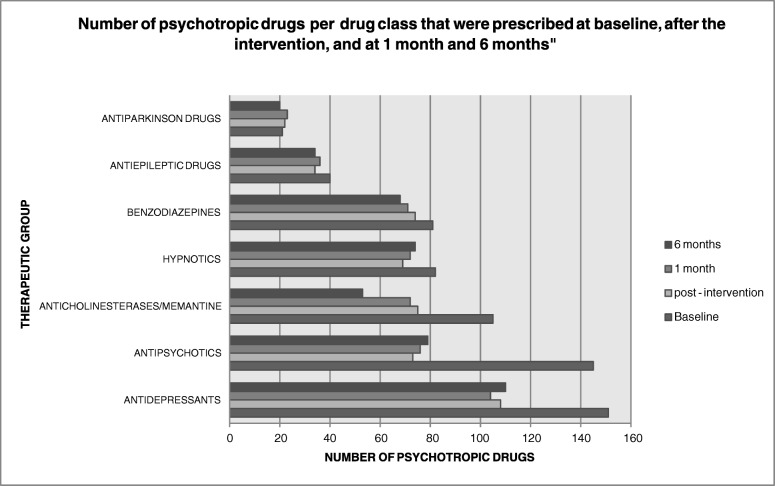
Number of psychotropic drugs per drug class that were deprescribed al baseline after the intervention, and at 1 month and 6 months

In the analysis according to drug class, antipsychotics were the class most frequently discontinued (49.66%), followed by acetylcholinesterase inhibitors and memantine (28.57%), and antidepressants (28.48%). However, hypnotics such as clomethiazole, hydroxyzine, and zolpidem were only reduced by 15.85%. The changes in psychotropic drug prescription by drug class along the study are shown in Fig. 2.

## Discussion

Patient-centered medication review, as conducted in this study with the support of dedicated therapy guidelines, achieved an overall 28% reduction in psychotropic drug prescription, which remained at an acceptable level at 6 months of follow-up for all the drug classes examined. The reductions were particularly marked for anticholinesterase inhibitors and antidepressant agents.

The decrease in prescribed psychotropic drugs achieved in this study was greater than that reported by other authors. In a clinical trial [25], Fossey et al. described a 19.1% reduction in nursing home residents with severe dementia. The differing values may be due to differences in the study designs and populations included. In the report by Fossey et al., deprescription was only contemplated for neuroleptic agents. Although neuroleptics were the drug class most commonly deprescribed in the present study, deprescription of any psychotropic drug was included, based on the patient’s response to treatment and current clinical status with regard to comorbidity, degree of dependence, and functionality. In a study by van der Spek [26], biannual medication review was applied to improve the appropriateness of the psychotropic drugs prescribed in nursing home patients with dementia. As the evaluation of the intervention differed from the one used here, the significant results obtained in improving the appropriateness of psychotropic drugs prescription cannot be compared with those of our study. However, the two studies coincide in the conclusion that a structured medication review by a multidisciplinary team can help to optimize psychotropic drug prescription in these patients. Other studies in nursing home patients (although not specifically dementia patients) [27, 28] have also provided evidence that multidisciplinary medication review can reduce the prescription of psychotropic drugs in institutionalized patients.

The fact that acetylcholinesterase inhibitors and memantine were the second most frequently deprescribed agents suggests a considerable percentage of patients with severe dementia—that is, showing a GDS stage of 7b or greater [29]. The study by Hogan and Strafford in patients with Alzheimer’s disease [30] emphasized the need for individualized assessment in the decision to deprescribe these drugs, even though there is consensus for their withdrawal in advanced stages of dementia.

At the 1-month follow-up, only 4 patients were restarted on previously discontinued psychotropic drugs, which could indicate that the patient-centered multidisciplinary approach used here was effective for optimizing and reducing prescription of psychotropic drugs. At 6 months, drugs were restarted in 22 patients, and in the case of anti-depressants, this may have been done in response to worsening of the depression symptoms. Although this clinical aspect was not specifically assessed in our study, it would coincide with the conclusion of Bergh et al. [31] that withdrawal of antidepressants in patients with dementia could worsen their depression symptoms, and that re-introduction of these drugs would be advisable. However, reintroduction of antipsychotic agents at 6 months could be due to the reappearance of symptoms once the patient had stabilized, as was suggested in the study by Devanand et al. [32]. This study concluded that discontinuation of antipsychotic agents can increase the probability that these symptoms will reappear. However, it is also important to note that reducing the exposure time to antipsychotic drugs can lower the probability of an adverse drug-related reaction [6]. The increase in the mean number of psychotropic drugs prescribed at 6 months following the intervention could have been due not only to reintroduction of some previous treatments, but also to the prescription of new treatments in order to control the appearance of new symptoms related to the evolution of the illness.

Following the intervention, a statistically significant reduction in prescribed psychotropic drugs occurred in all nursing home except one, in which the decrease was smaller and non-significant. The mean number of drugs used per patient in this center did not differ from that of the other nursing homes, but 82% of the patients were receiving 3 or fewer drugs, and our study results showed that prescription changes were more prevalent in patients receiving 4 or more drugs. In addition, 39% of the psychotropic drugs used were antiepileptic agents or specific medication for treating dementia. Along the intervention, antiepileptics were the drug class showing the smallest reduction, and dementia-specific treatment was generally considered appropriate and not a candidate for discontinuation. These factors may have played a part in the smaller prescription decrease observed.

Regarding the limitations of the study, we should note that it is a pre/post study with no control group. In selecting this design, we took into consideration the growing evidence of psychotropic drug-related adverse effects in patients with dementia [14–17] [29, 30]. The design was considered to be a pragmatic primary health care approach. When the intervention was being considered, we saw that the prevalence of psychotropic drug use in our dementia patients was higher than that described by Gustafsson [8] and other authors [9, 10]. We believed that the intervention would improve the routine management of this population, and a control group was not contemplated so that all individuals participating could benefit from it. As all dementia patients eligible for the intervention were included, we did not calculate the sample size a priori. However, at completion of the study, a power analysis was performed using the G*power program [24], which yielded a statistical power greater than 80%.

Another limitation was that no further clinical evaluations were carried out after the initial patient assessment; only medication-related changes were recorded. However, we were able to show that deprescription decisions remained in the sample over some months. Furthermore, selection of the nursing homes to be included in the study was not randomized. The intervention was proposed in all large nursing homes in the catchment area of the primary care team, and some chose not to participate. All the candidate nursing homes were of a general nature (ie, they were not centers specialized in certain conditions) and there were no significant differences in the age of the residents between those that participated or not in the study. However, the participating centers cannot be considered representative of all nursing homes in our setting. Finally, the medication review took into account the patient’s health status, frailty, and prognosis, but the patient did not take part in the treatment decisions.

## Conclusion

In conclusion, an intervention based on dedicated, evidence-based therapeutic guidelines designed by a multidisciplinary team and implemented by consensus, together with a patient-centered clinical medication review, led to a reduction in prescription of psychotropic drugs in institutionalized dementia patients. As this illness and its associated symptoms may vary considerably over time, it is important to review the patient’s medication on an individual basis periodically to ensure that it is appropriate according to benefit/risk criteria.

## Additional files

Additional file 1: Therapeutic guideline for treating BPSD used in this study (written in Catalan). (PDF 306 kb)

Additional file 2: Brief summary of the consensus guidelines and development of the pharmacological review intervention for the management of BPSD. (PDF 46 kb)

## Abbreviations

ATC: The Anatomical Therapeutic Chemical Classification System of the World Health Organization
BPSD: Behavioral and psychological symptoms of dementia
GDS: Global Dementia Scale
GP: General practitioner
ICS: *Institut Català de la Salut (*Catalan Health Institute)
IDIAPJGol: *Fundació Institut Universitari per a la recerca a l'Atenció Primària de salut Gol* i Gurina (Jordi Gol Primary Care Research Institute)
NH: Nursing home
PD: Psychotropic drugs
SD: Standard deviation
SMD: Severe mental disorder
