# Supplementary material for: Consensus and evidence-based medication review to optimize and potentially reduce psychotropic drug prescription in institutionalized dementia patients
Source: BMC Geriatr. 2019 Jan 8;19:7. doi: 10.1186/s12877-018-1015-9 (PMC6323667; doi:10.1186/s12877-018-1015-9)
Supplement: Supplementary file 1 — Therapeutic guideline for treating BPSD used in this study (written in Catalan). (PDF 306 kb) [file 12877_2018_1015_MOESM1_ESM.pdf]

Guia de recomanacions terapèutiques per l'abordatge dels Símptomes Psicològics i Conductuals relacionats amb la Demència (SPCD)

Abril 2012

Institut Català de la Salut
Servei d'Atenció Primària
Barcelonès Nord i Maresme

CONSORCI SANITARI DEL MARESME

COORDINACIÓ

Espinosa, Mª Carmen (Geriatra, CSdM)

Massot, Mireia (Farmacèutica, ICS)

AUTORS

Cristòfol, Ramon (Geriatra, CSdM)

López Faixó, David (Farmacèutic, CSdM)

Peláez, Jordi (Farmacèutic, CatSalut)

Rodríguez Darriba, Mercè (Farmacèutica, CatSalut)

Sanz, Pilar (Neuròloga, CSdM)

Sehabiaga, Pedro (Psiquiatre, CSMA-Maresme Sud - Comunidad Terapéutica del Maresme)

Tria, Carles (Metge de Família, CSdM)

Tristany, Montserrat (Metgessa de Família, ICS)

JUSTIFICACIÓ

La demència es una malaltia degenerativa que cursa amb deteriorament de la memòria i de al menys una del resta de les funcions cognitives, produint discapacitat ocupacional o social.

Les demències cursen molt freqüentment amb símptomes neuropsiquiàtrics, anomenats també símptomes psicològics i conductuals associats a la demència (SPCD). Aquests SPCD produeixen un gran estrés en el pacient i en el cuidador, afectant a la qualitat de vida de tots ells.

La presència de símptomes com agitació, irritabilitat, trastorns de la son, il·lusions, al·lucinacions o agressió són una de las consultes més freqüents dels pacients afectats de demència, on, a vegades són desencadenants de ingressos hospitalaris o en residència geriàtrica[1].

Durant un període de cinc anys, com a mínim el 90% desenvolupa algun símptoma que en el 85% de los casos, pot arribar a tenir implicacions clíniques importants[2].

Diferents estudis han demostrat l'existència d'una relació entre el grau de deteriorament cognitiu i la freqüència i severitat d'aquests símptomes, o al menys per alguns d'aquests símptomes. La història natural de la demència revela que pacients amb símptomes psicòtics presenten un deteriorament cognitiu més ràpid.

Els símptomes psicòtics són més freqüents en las fases moderades i severes de la malaltia. Per el contrari, la relació de la depressió amb l'estadi de la malaltia és menys clara, però alguns estudis apunten que hi hauria una relació inversa, present en les fases primerenques de la malaltia i absent en las fases més severes. No obstant, aquest fet també pot ser degut a la problemàtica del diagnòstic de la depressió en estadiatges avançats de la malaltia, que a una reducció de la prevalència[3].

VALORACIÓ PER ATENCIÓ PRIMÀRIA

La valoració i el tractament tant farmacològic com no farmacològic des de l'atenció primària es realitzarà per agrupacions de símptomes.

En la demència els SPCD s'agruparen de la següent manera:

GRUPS DE SÍMPTOMES [1,4]

1. Apatia
2. Agressivitat

- resistència agressiva, agressivitat física i verbal.

3. Agitació/agitació psicomotora

- caminar incessantment i/o sense sentit, seguir a altres, inquietud, accions repetitives i trastorn circadià.

4. Depressió

- tristesa, plor, desesperança, culpa, ansietat.

5. Psicosis

- al·lucinacions, deliris.
- *falses identificacions- no es un trastorno psicòtic.*

6. Trastorns de la son

SÍMPTOMES DE DIFÍCIL CONTROL [5]

Davant d'una consulta per un SPCD, hem de tenir en compte un aspecte fonamental. No tots els símptomes podran ser tractats amb un medicament. Quan aquests símptomes, sí poden ser tractats amb un medicament, és necessària la revaloració constant del pacient, ja que el seu estat degeneratiu varia ràpidament i amb aquest, els SPCD associats, cosa que fa imprescindible la revaloració de la necessitat o no de tractament al llarg del temps.

En la següent taula es relaciona els símptomes que generalment no responen al tractament i els símptomes que potser puguin respondre.

Comportaments que generalment no responen al tractament	Comportaments que potser responguin al tractament
‣ Deambulació erràtica.<br>‣ Orinar i defecar en llocs inapropiats.<br>‣ Vestir-se i desvestir-se en llocs inapropiats.<br>‣ Comportaments repetitius.<br>‣ Amagar/acumular.<br>‣ Pica (menjar coses no comestibles)<br>‣ Aïllament.<br>‣ Arrancar-se la roba.	‣ Agressivitat física.<br>‣ Agressivitat verbal.<br>‣ Ansietat, inquietud psicomotriu.<br>‣ Tristesa.<br>‣ Crits.<br>‣ Anorèxia.<br>‣ Apatia.<br>‣ Trastorn de la son.<br>‣ Deambulació erràtica amb agitació i agressivitat.<br>‣ Comportaments vocals repetitius.<br>‣ Il·lusions/al·lucinacions.<br>‣ Comportament sexual inapropiat amb agitació

CONSIDERACIONS GENERALS PER L'ABORDATGE NO FARMACOLÒGIC I FARMACOLÒGIC DELS SPCD[6-22]

Identificar **SÍMPTOMA DIANA** a tractar

Aquest símptoma pot ser un **EFECTE ADVERS** a un medicament que estigui prenent?

Sí

Valorar la **SUSPENSIÓ o SUBSTITUCIÓ** d'aquest medicament tenint en compte el balanç benefici/risc

Seguiment:

- **SUSPENSIÓ:** monitoratge del pacient per si desaparició del símptoma diana i/o aparició de nou el problema de salut.
- **SUBSTITUCIÓ** : monitoratge del pacient per si desaparició del símptoma diana

No

Existeix un **DETONANT** de la conducta? o Existeix algun **problema orgànic** que el pugui causar?

Sí

RESOLDRE el detonant o **TRACTAR** el problema de salut base

No

Existeix un **ABORDATGE NO FARMACOLÒGIC**?

Sí

APLICAR l'abordatge NO FARMACOLÒGIC

SEGUIMENT:

Monitorar efecte de l'abordatge no farmacològic

Abordatge **NO EFICAÇ i símptoma molt DISRUPTIU**

No

Consideracions GENERALS en cas de ser necessari un TRACTAMENT FARMACOLÒGIC:

1. **NECESSITAT**: És necessari un medicament per tractar aquest símptoma?
2. **SIMPLIFICACIÓ TERAPÈUTICA**: Si el pacient presenta dos o més SPCD, es pot tractar al pacient amb UN SOL FÀRMAC? (Annexe 1: tractament per Clústers).
3. **BAIXA DOSI**: És la **DOSI MÍNIMA** eficaç? Necessita un **AJUSTAMENT** de dosi per edat, sexe o patologia concomitant?
4. **SEGURETAT**: Aquest medicament presenta **EFECTES ADVERSOS** amb més freqüència en gent gran? Aquest medicament és potencialment **INAPROPIAT** per la gent gran?
5. **REVISIÓ de la MEDICACIÓ: Quins criteris i amb quina periodicitat es revisarà l'eficàcia d'aquest medicament? Aquesta revisió és IMPRESCINDIBLE per aquells símptomes que potser responguin a un tractament farmacològic.**

Recomanacions abordatge NO farmacològic

- Intervenció precoç per evitar l'escalada del símptoma.
- Mantenir la calma, abordar-lo amb suavitat, utilitzar un to de veu baix i mirar-lo als ulls.
- Explicar en cada moment al pacient què se li va a fer, amb paraules senzilles.
- Proveir una rutina al pacient (hora de llevar-se i anar-se'n a dormir, etc.).
- Simplificar les tasques, per que pugui realitzar-les.
- Reduir l'estimulació excessiva, inclusiu el soroll de la Televisió, ràdio, conversacions,...
- Buscar un entorn segur: retirar catifes, terres relliscosos, obstacles, cordons elèctrics,...
- Utilitzar música, preferiblement la que agradi al pacient, durant activitats com menjar i bany.
- Mantenir la llum per la tarda/nit per reduir la confusió i la inquietud nocturna.
- La contenció mecànica **és inacceptable**.

Consideracions a la prescripció de antipsicòtics[9]

- Revisar els factors de risc cardiovascular i/o malalties cardiovasculars.
- Escollir el fàrmac més indicat i iniciar a dosis baixes.
- Explicar els possibles afectes positius i negatius de la medicació.
- Discutir la seva prescripció amb el representant/responsable del pacient.
- Indicats los atípics.
- Es pauten quan l'agitació/simptomatologia psicòtica no ha respost al maneig ambiental i la intensitat és important.
- L'ús d'antipsicòtics s'ha d'iniciar a la mínima dosi disponible amb un seguiment estret dels pacients amb l'objectiu de mantenir-los el mínim temps possible a la mínima dosi eficaç.
- Els antipsicòtics[16,17] disposen de la millor evidència per al tractament a curt termini de l'agressivitat i l'agitació amb idees delirants, però no han demostrat benefici en el tractament dels símptomes no agressius de l'agitació.
- Mantenir durant un període d'un mes aproximadament i suspendre si la resposta o el beneficio no es clar.
- Si la resposta és bona, re considerar el seu manteniment a las 6-12 setmanes de la seva prescripció.
- El temps recomanat d'espera per al canvio de fàrmac si la resposta és inadequada és de 5 a 7 dies, mantenint-se de 1 a 3 mesos una vegada aconseguit el control de símptomes i intentant posteriorment una retirada paulatina el més precoç possible, segons intensitat i recurrències del símptoma.

Els criteris STOPP/START[23]

S'han publicat recentment uns criteris, anomenats STOPP-START (Screening Tool of Older Person's potentially inappropriate Prescriptions/Screening Tool to Alert doctors to the Right, i.e. appropriate, indicated Treatment), nascuts a Irlanda, on el seu desenvolupament clínic ha estat assumit por l'European Union Geriatric Medicine Society. Aquests criteris, organitzats per sistemes fisiològics, recullen els errors més comuns de tractament i omissió en la prescripció i són fàcils de relacionar amb els diagnòstics actius i la llista de fàrmacs que apareixen en les històries clíniques informàtiques dels pacients. Consten, en realitat, de dos grups de criteris: els STOPP (per la paraula anglesa ‘‘parar’’) i els START (per ‘‘començar’’).

Els criteris STOPP, com ja s'ha mencionat anteriorment, s'organitzen per sistemes fisiològics. Per el desenvolupament d'aquesta Guía terapèutica dels SPCD, només exposarem els criteris STOPP relacionats amb la medicació necessària per tractar aquests símptomes.

1. **Sistema Nerviós central i psicofàrmacs:**
   a. Antidepressius tricíclics (ATC) amb demència (*risc d'empitjorament del deteriorament cognitiu*).
   b. ATC amb glaucoma (*possible exacerbació del glaucoma*).
   c. ATC amb trastorns de la conducció cardíaca (*efectes pro arrítmics*).
   d. ATC amb estrenyiment (*possible empitjorament de l'estrenyiment*).
   e. ATC amb un opiaci o un Antagonista del calci (*risc de estrenyiment greu*).
   f. ATC amb prostatisme o amb antecedents de retenció urinària (*risc de retenció urinària*).
   g. Us prolongat (i.e. més de 1 mes) de benzodiazepines de vida mitja llarga (com clordiazepòxid, flurazepam, nitrazepam, clorazepat) o benzodiazepines amb metabòlits de llarga acció (com diazepam) (*risc de sedació prolongada, confusió, trastorns de l'equilibri, caigudes*).
   h. Us prolongat (i.e. més de 1 mes) de neurolèptics com hipnòtics a llarg termini (*risc de confusió, hipotensió, efectes extrapiramidals, caigudes*).
   i. Us prolongat de neurolèptics (i.e. més de 1 mes) en el parkinsonisme (*es probable que empitjorin els símptomes extrapiramidals*).
   j. Fenotiazines en pacients amb epilèpsia (*poden baixar l'umbral convulsiu*).
   k. Anticolinèrgics per tractar els efectes secundaris extrapiramidals dels neurolèptics (*risc de toxicitat anticolinèrgica*).
   l. ISRS amb antecedents d'hiponatrèmia clínicament significativa (*hiponatrèmia inferior a 130mmol/l no iatrogènica en els dos mesos anteriors*).

m. Us prolongat (més de 1 setmana) d'antihistamínics de primera generació, i.e. difenhidramina, clorfeniramina, ciclizina, prometazina (*risc de sedació i efectes secundaris anticolinèrgics*).

2. **Fàrmacs que afecten negativament als propensos a caure (1 o més caigudes en els últims tres mesos)**

   a. Benzodiazepines (*sedants, poden reduir el sensori, deteriorant l' equilibri*).

   b. Neurolèptics (*poden causar dispràxia de la marxa, parkinsonisme*).

   c. Antihistamínics de primera generació (*sedants, poden reduir el sensori*).

   d. Vasodilatadors dels que se sap que poden causar hipotensió en aquells amb hipotensió postural persistent, i.e. descens recurrent superior a 20mmHg de la pressió sistòlica (*risc de síncopes, caigudes*).

   e. Opiacis a llarg termini en aquells amb caigudes recurrents (*risc de somnolència, hipotensió postural, vertigen*).

1. Tractament de l'Apatia com a SPCD:

Definició: Falta de motivació amb comportament i cognició disminuïts en quant a plantejament d'objectius i un reduït compromís emocional. Ha de ser considerada com significativa, quan representa un canvi en el comportament previ del pacient i/o causa una important discapacitat funcional.

Recomanacions per l'inici del tractament en l'apatia:

1. Apatia que genera un deteriorament funcional.
2. Potencial de millor en la qualitat de vida (o canvi en la qualitat de vida) del pacient.
3. Angústia/carga dels cuidadors i/o familiars, encara que depen de les expectatives del cuidador, la personalitat pre-mòrbida del pacient i l'entorn en el que està vivint el pacient. Ex. Els membres d'una família poden estar més angustiats si es tracta d'una persona activa que es torna apàtica, mentre que en una residència l'apatia pot ser més ben tolerada.

Algoritme d'abordatge de l'apatia:

Els medicaments calci-antagonistes, la pentoxifilina i els antiepilèptics no han demostrat eficàcia en el tractament de l'apatia en pacients amb demència.

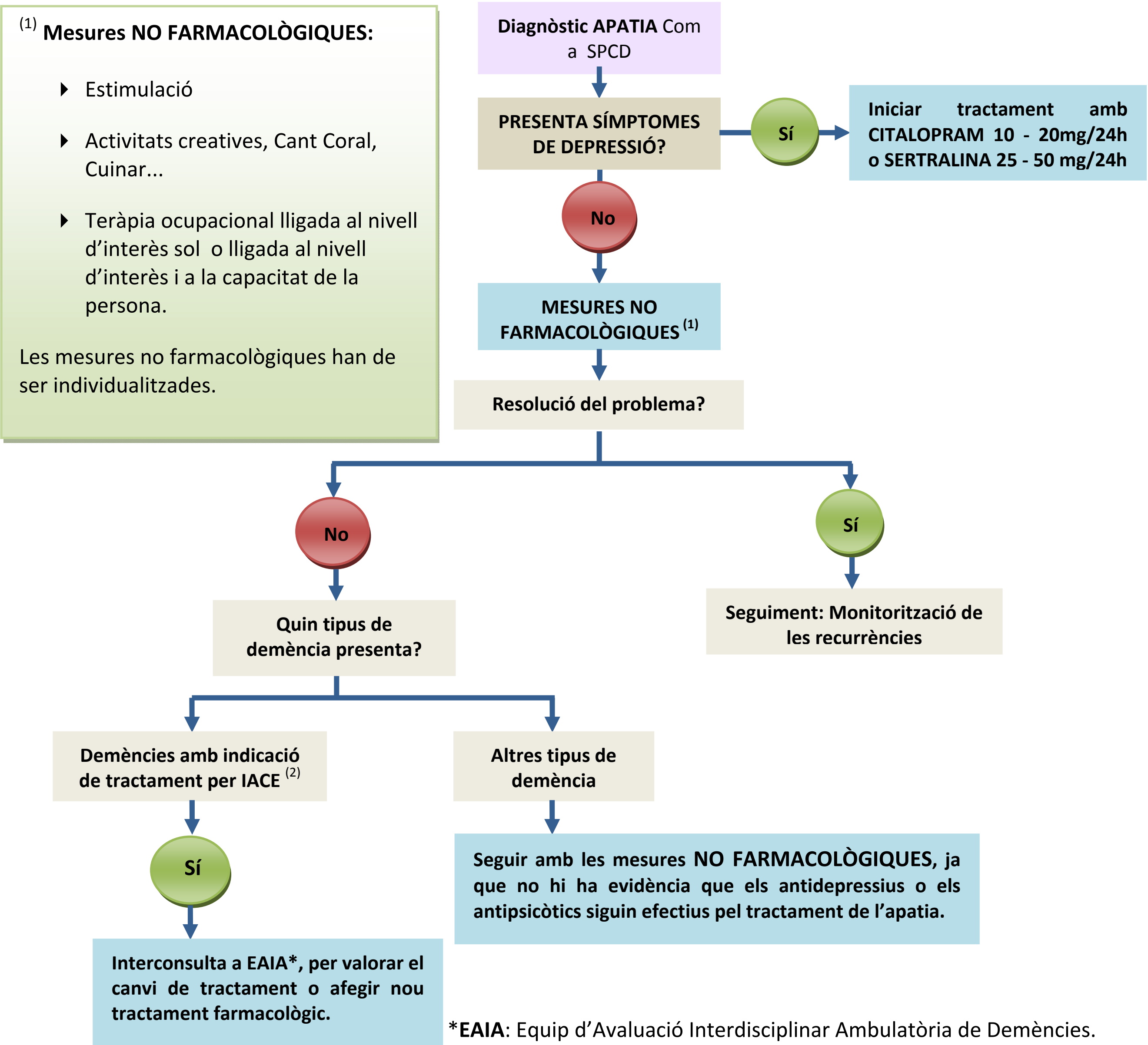

***EAIA**: Equip d'Avaluació Interdisciplinar Ambulatòria de Demències.

[2] **Criteris tractament amb Inhibidors de l'Acetilcolinesterasa (IACE)** Instrucció 1/2011 Direcció General de Regulació, Planificació i Recursos Sanitaris Departament de Salut.

I. Malalts amb diagnòstic de "malaltia d'Alzehimer " segons els criteris DSM-IV.
II. Malalts amb diagnòstic de "malaltia d'Alzehimer probable" segons els criteris NINCDS-ADRDA o amb diagnòstic de "malaltia d'Alzehimer possible" (Demència per cossos de Lewi, mixta o associada a malaltia de Parkinson) en que s'acrediti la necessitat del tractament mitjançant un informe que ho justifiqui.

2. Tractament de l'Agressivitat com a SPCD:

Definició: Comportament o intenció de fer mal, ofendre a algú mitjançant insults, comentaris o físicament per cops, lesions o violència.

Algoritme d'abordatge de l'agressivitat:

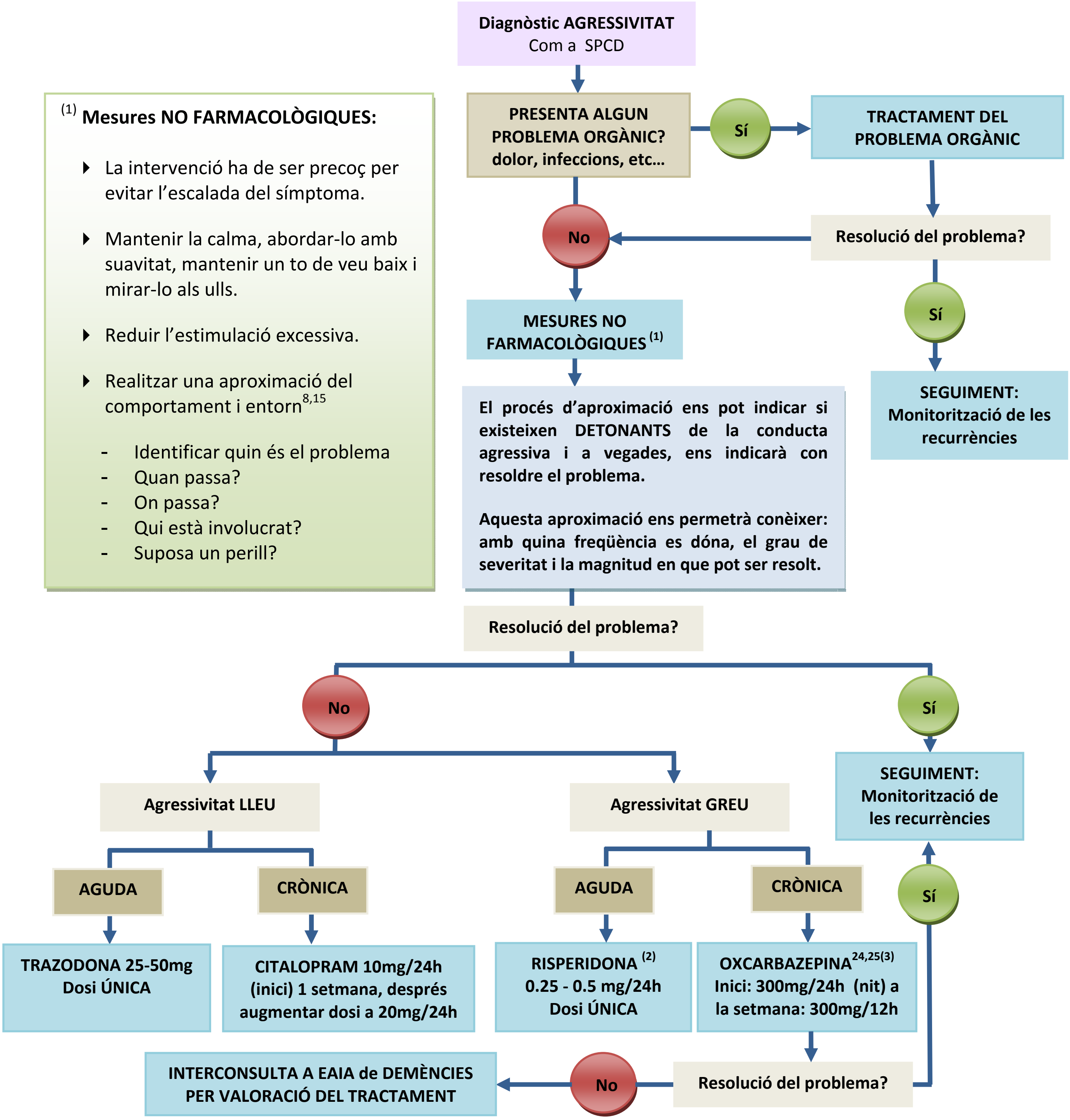

[2] Si no control símptomes REPETIR DOSI (dosi màxima 2mg/24h). Els pacients amb la malaltia de Parkinson considerar **QUETIAPINA** 12.5 – 25 mg/24h Dosi ÚNICA.

[3]Cal tenir la precaució del risc d'Hiponatrèmia. Si no aconseguim control de símptomes: AFEGIR RISPERIDONA 0.25 a 0.5 mg/dia (dosi màxima de 2mg) mantenir de 1 a 3 mesos, una vegada arribat al control de símptomes retirar el fàrmac paulatinament. Els pacients amb la malaltia de Parkinson considerar **QUETIAPINA** 12.5 – 25 mg/24h,dosi màxima 100 mg/dia. Presenta un efecte hipotensor més marcat que la Risperidona. La retirada es planteja igual que la Risperidona. En cas d'estar tractat prèviament amb ISRS, es recomana la retirada d'aquest fàrmac per tal no associar psicofàrmacs en la gent gran i disminuir els efectes adversos.

3. Tractament de l'Agitació/agitació psicomotora com a SPCD:

Definició: Excessiva activitat motora associada a un sentiment d'estrés interior.

Es proposa la descripció dels diferents tipus d'agitació que recull Cohen-Mansfiel en la seva escala[26] (annex 2).

Per al tractament adequat de l'agitació és imprescindible **realitzar un diagnòstic diferencial de l'agitació**, ja que en funció de l'origen d'aquest símptoma el tractament, tant farmacològic com no farmacològic diferirà substancialment.

En la gran majoria dels casos d'agitació com a SPCD en gent gran presenten un **SÍNDROME CONFUSIONAL AGUT** , on **la base del tractament radica en identificar la causa de l'agitació** i resoldre el problema base, deixant el tractament farmacològic per aquells casos en que l'agitació sigui causa d'un Trastorn neuro-psiquiàtric.

SÍMPTOMES SÍNDROME CONFUSIONAL AGUT:
- Deteriorament sobtat
- Curs fluctuant
- Al·lucinacions
- Alteracions de la son

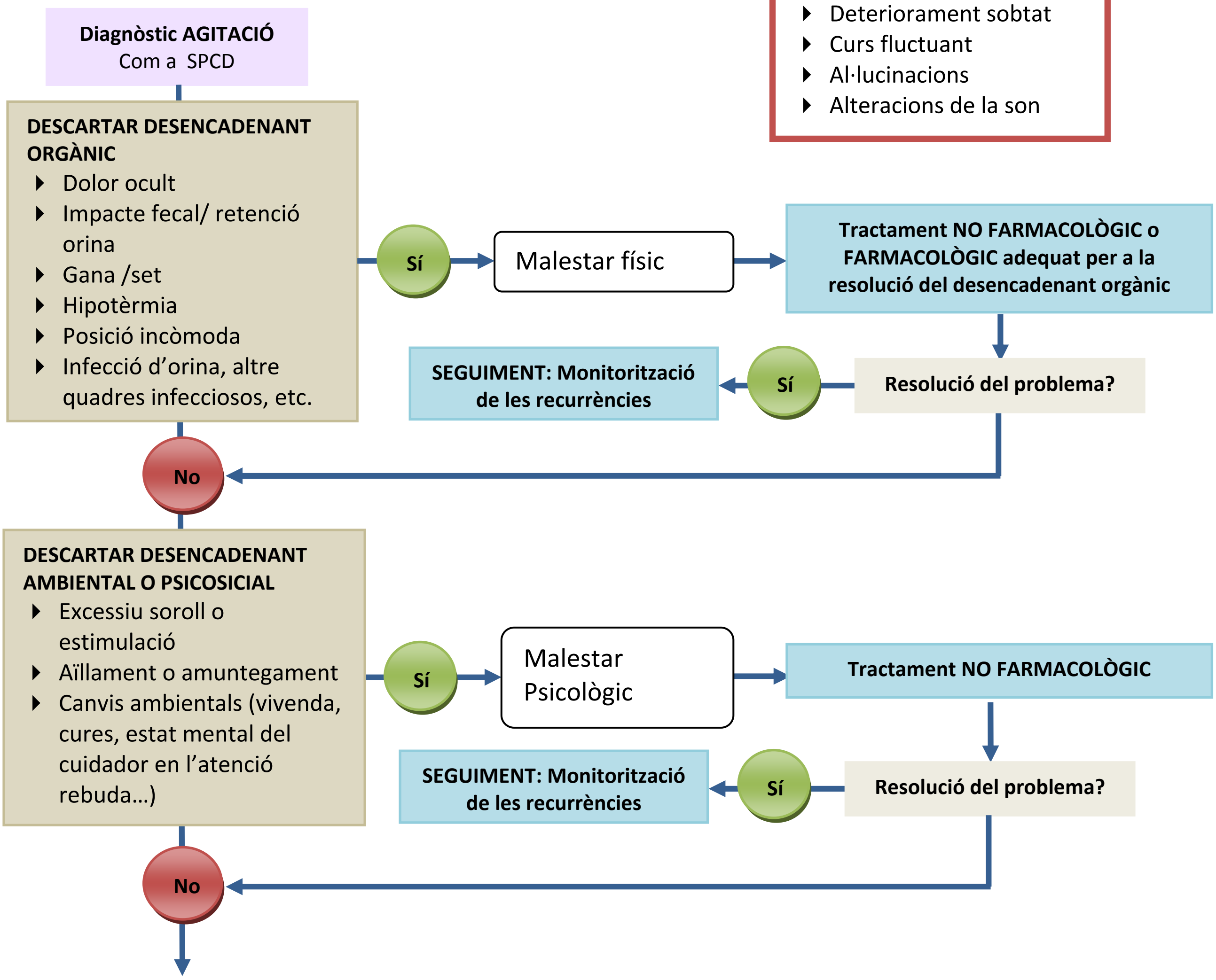

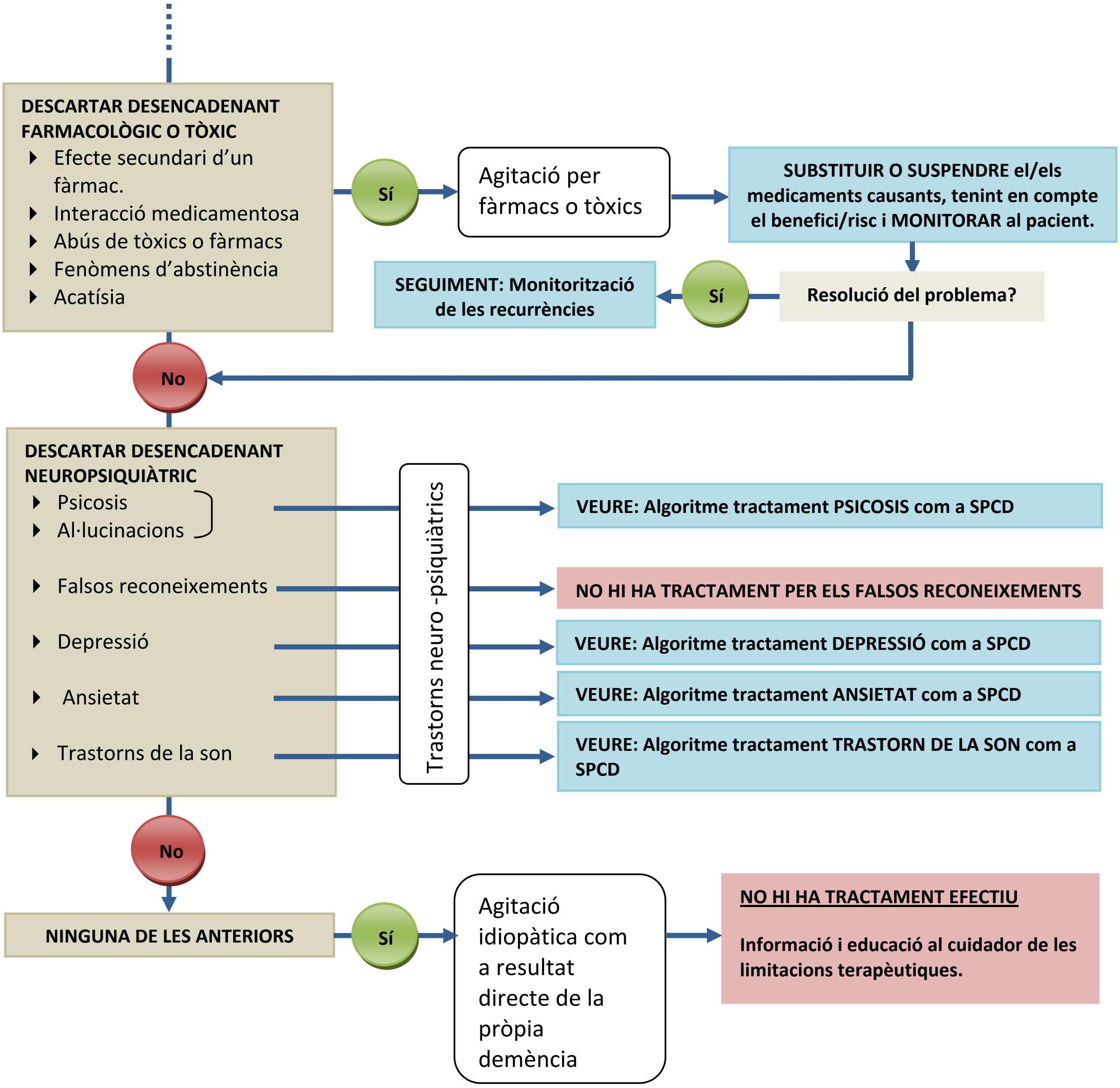
DESCARTAR DESENCADENANT FARMACOLÒGIC O TÒXIC
Efecte secundari d'un fàrmac.
Interacció medicamentosa
Abús de tòxics o fàrmacs
Fenòmens d'abstinència
Acatísia
Sí
Agitació per fàrmacs o tòxics
SUBSTITUIR O SUSPENDRE el/els medicaments causants, tenint en compte el benefici/risc i MONITORAR al pacient.
Resolució del problema?
Sí
SEGUIMENT: Monitorització de les recurrències
No
DESCARTAR DESENCADENANT NEUROPSIQUIÀTRIC
Psicosis
Al·lucinacions
Falsos reconeixements
Depressió
Ansietat
Trastorns de la son
Trastorns neuro -psiquiàtrics
VEURE: Algoritme tractament PSICOSIS com a SPCD
NO HI HA TRACTAMENT PER ELS FALSOS RECONEIXEMENTS
VEURE: Algoritme tractament DEPRESSIÓ com a SPCD
VEURE: Algoritme tractament ANSIETAT com a SPCD
VEURE: Algoritme tractament TRASTORN DE LA SON com a SPCD
No
NINGUNA DE LES ANTERIORS
Sí
Agitació idiopàtica com a resultat directe de la pròpia demència
NO HI HA TRACTAMENT EFECTIU
Informació i educació al cuidador de les limitacions terapèutiques.

4. Tractament de la Depressió com a SPCD:

Definició: Trastorn de l'estat d'ànim caracteritzat per tristesa i incapacitat d'experimentar plaer (anhedonia). Es manifesta a través d'expressions i sentiments d'inutilitat, desesperança, desvalorització, o de culpabilitat injustificats; pensaments recurrents de mort, poca capacitat de concentració, augment de la fatiga i pèrdua d'energia.

El diagnòstic és clínic, on s'ha de descartar prèviament, organicitat, causes tòxiques, farmacològiques, que permetin deixar en clar els criteris per parlar de Depressió.

Algoritme d'abordatge de la depressió:

(1) **Mesures NO FARMACOLÒGIQUES:**

- Exercici
- Estimulació lumínica
- Proposar activitats lúdiques terapèutiques.
- Resulta útil poder contar amb recolzament psicoterapèutic a curt termini orientat a reconduir pensaments negatius, distorsionats, o si el pacient no pot o no desitja tomar medicació.

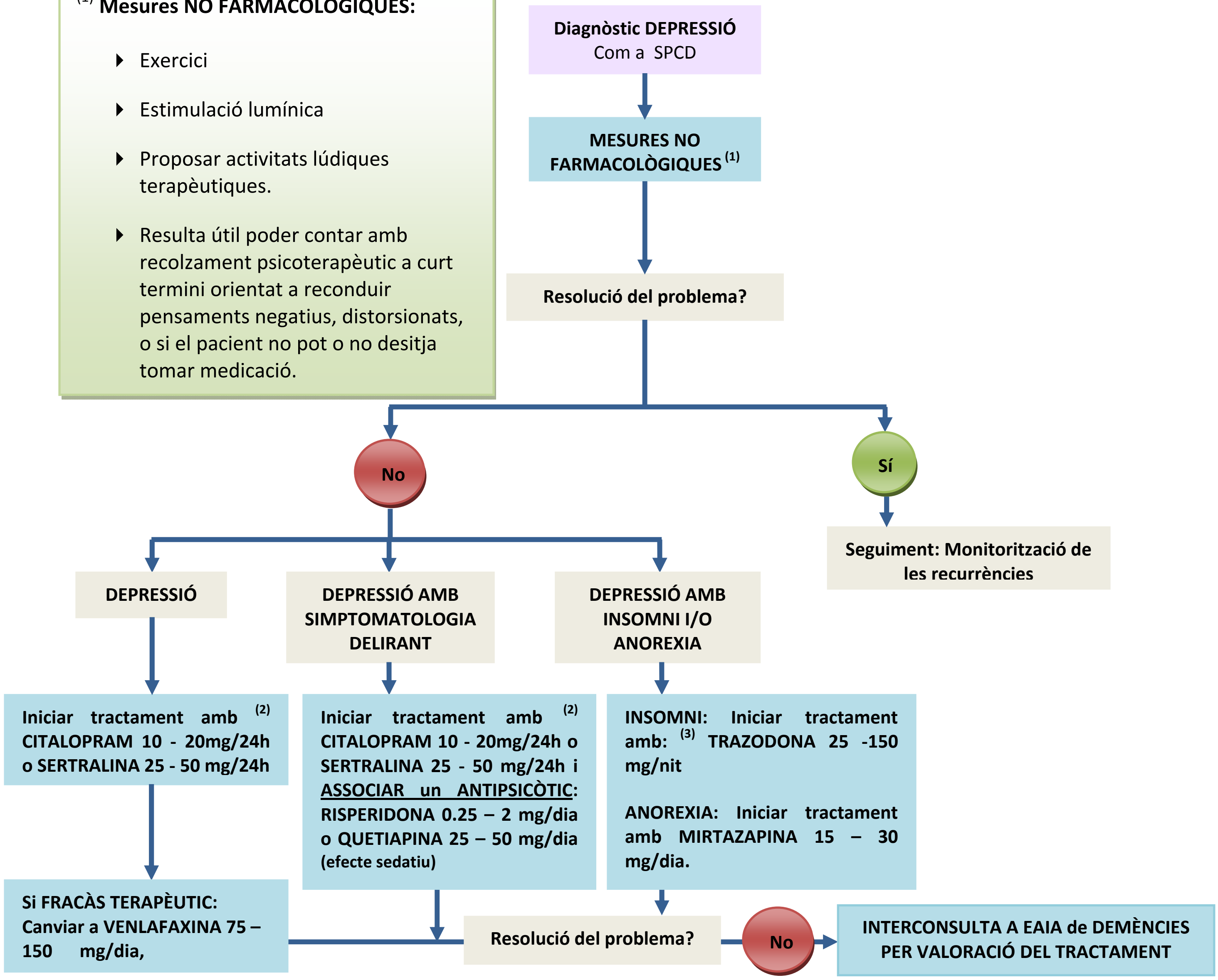

(2) L'inici d'acció dels antidepressius és de 9 a 12 setmanes després d'haver iniciat el tractament. La durada del tractament amb un antidepressiu hauria de ser de 6 a 12 mesos mínim, segons criteri clínic, als efectes de reinstaurar un adequat funcionament del pacient, disminuir la morbilitat i la mortalitat, evitant les recaigudes, sempre que sigui possible.

(3) Recordar que a menor dosi, la Mirtazapina té un major efecte sedant. Un dels efectes adversos que presenta és l'augment de la gana, on haurem de fer un balanç benefici/risc en funció del pacient.

5. Tractament de l'Ansietat com a SPCD:

Definició: Resposta emocional complexa amb una percepció exagerada i injustificada d'amenaça, mals pressentiments i pensaments de mort imminent, que provoca una activació biològica dirigida a reaccionar front a aquesta percepció.

Algoritme d'abordatge de l'ansietat:

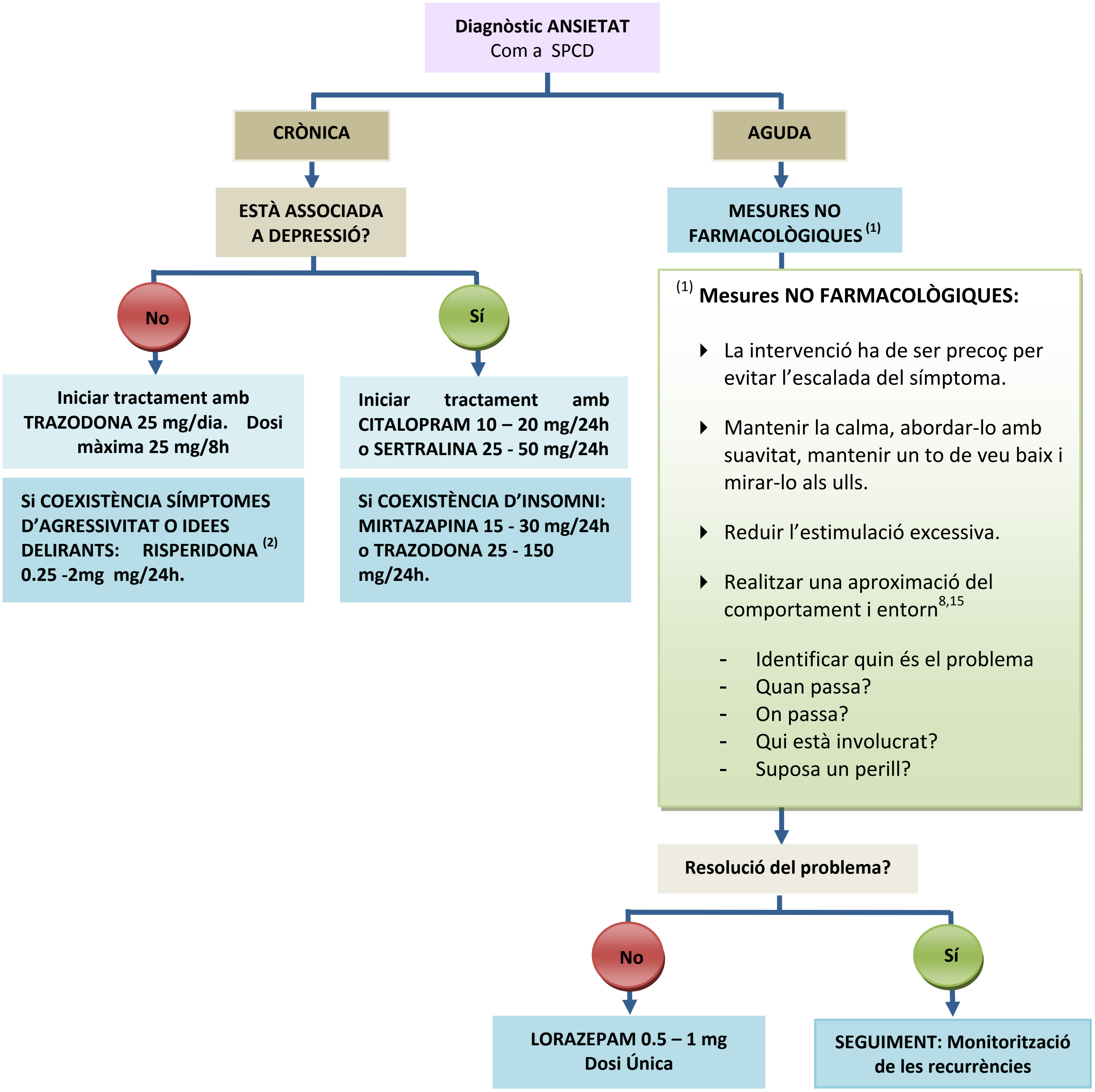

[2] Els pacients amb la malaltia de Parkinson considerar **QUETIAPINA** 12.5 –50 mg/24.

6. Tractament de la Psicosi com a SPCD:

Definició:

<u>Deliris</u>- falses creences basades en inferències incorrectes sobre la realitat externa, que el malalt manté firmament, encara que s'evidenciï el contrari.

<u>Al·lucinacions</u>- percepcions sensorials que donen la mateixa sensació de realitat que una veritable experiència sensorial però que es donen sense estimulació de l'òrgan sensorial rellevant.

<u>Il·lusions</u>- males interpretacions dels estímuls visuals externs. Es distingeixen de les al·lucinacions en que són percepcions distorsionades en lloc de percepcions anormals espontànies.

Algoritme d'abordatge de la psicosi:

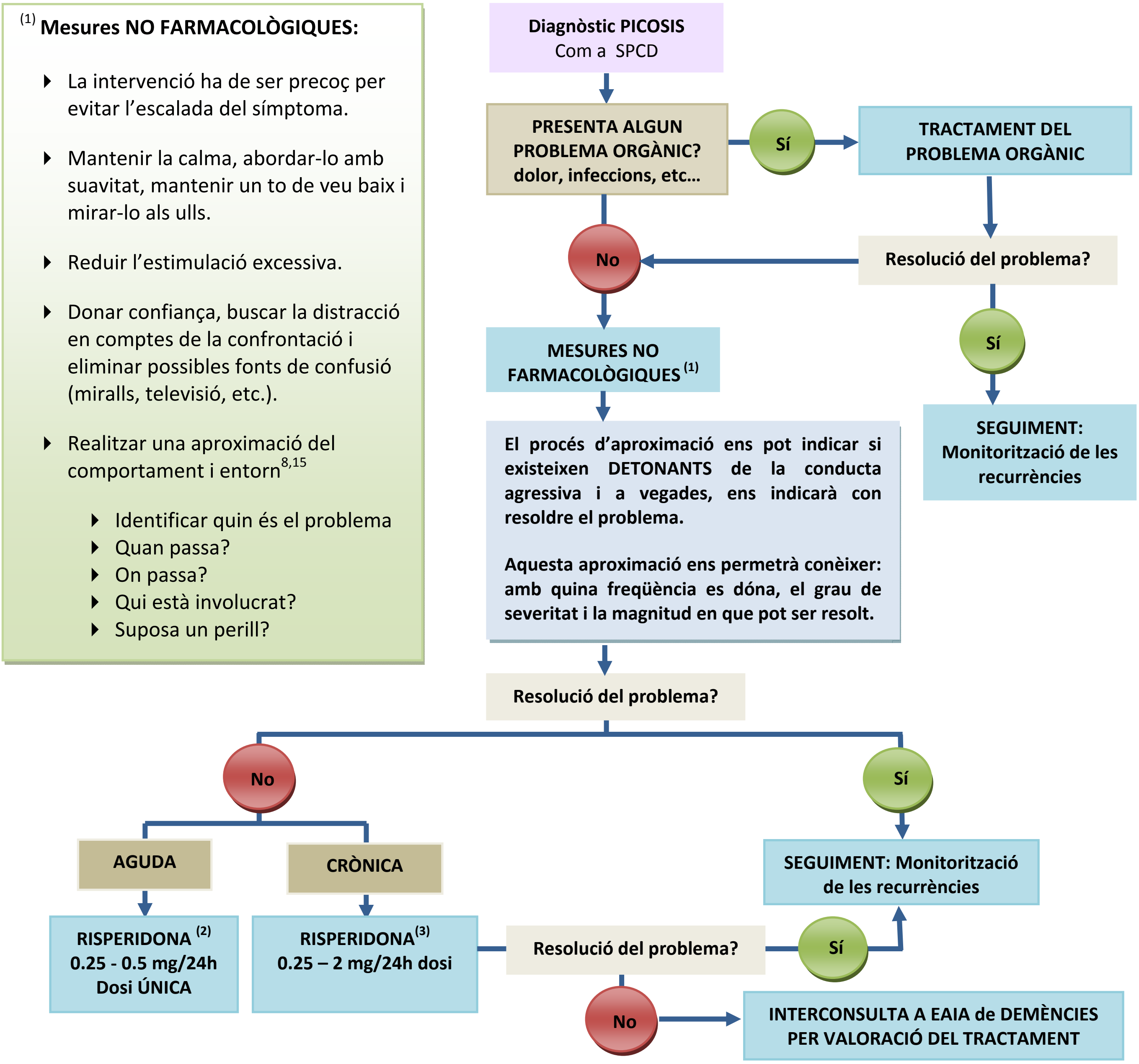

(2) Els pacients amb la malaltia de Prakinson considerar **QUETIAPINA** 12.5 – 25 mg/24h Dosi ÚNICA.

(3)Mantenir de 1 a 3 mesos, una vegada arribat al control de símptomes retirar el fàrmac paulatinament. Els pacients amb la malaltia de **Parkinson** o presència de **SÍMPTOMES EXTRAPIRAMIDALS** considerar **QUETIAPINA** 12.5 – 25 mg/24h,dosi màxima 100 mg/dia. Presenta un efecte hipotensor més marcat que la Risperidona. La retirada es planteja igual que la Risperidona. En cas d’estar tractat prèviament amb ISRS, es recomana la retirada d’aquest fàrmac per tal no associar psicofàrmacs en la gent gran i disminuir els efectes adversos.

7. Tractament dels Trastorns de la son com a SPCD:

Definició: dificultat per conciliar la son, la baixa qualitat d'aquesta o la seva fragmentació.

Algoritme d'abordatge dels trastorns de la son:

(1) **Mesures NO FARMACOLÒGIQUES:**

- Higiene de la son:
  - Regularitat a l'hora d'anar al llit i llevar-se
  - Limitar el temps diari de llit al temps necessari de son, 7-8 hores
  - Evitar migdiades, mai al llit
  - Evitar el consum de substàncies amb efecte estimulador del SNC
  - Fer exercici físic moderat, mai a les darreres hores del dia.
  - Mantenir condicions ambientals adequades per dormir: temperatura, sorolls, llum, matalàs..
  - Sopar moderat 2-3 hores abans anar al llit
  - Utilitzar el llit només per dormir
  - Banyar-se amb l'aigua a temperatura corporal, per el seu efecte relaxant
- Estimulació durant el dia
- Evitar estimulació excessiva durant la tarda

Diagnòstic TRASTORN DE LA SON com a SPCD

PRESENTA ALGUN PROBLEMA ORGÀNIC? dolor, infeccions, etc…

Sí → **TRACTAMENT DEL PROBLEMA ORGÀNIC** → **Resolució del problema?**
- **No** → MESURES NO FARMACOLÒGIQUES
- **Sí** → **SEGUIMENT: Monitorització de les recurrències**

No → **MESURES NO FARMACOLÒGIQUES (1)** → **Resolució del problema?**

- **Sí** → **SEGUIMENT: Monitorització de les recurrències**
- **No** →
  - **INSOMNI** → **CLOMETIAZOL (2) 192 – 384 mg/nit o TRAZODONA 50 -150 mg/nit**
  - **INSOMNI + ANSIETAT I/O DEPRESSIÓ** → **TRAZODONA 50-150mg o MIRTAZAPINA 15-30 mg/24h**
  - **INSOMNI + AGRESSIVITAT O SIMP PSICÒTICS** → **QUETIAPINA 12,5 -50mg/24h o RISPERIDONA 0,25 -2mg/24h.**

(2) Per la seva tolerància valorar durada curta tractament

TRACTAMENTS PER L' INSOMNI:

Pel tractament de l' Insomni, **no es recomana l'addició de més d'un fàrmac psicoactiu**. La recomanació es fonamenta en l'aplicació de mesures higièniques de la son més un únic tractament farmacològic, en cas de fracàs terapèutic es recomana la suspensió del fàrmac i la instauració d'un nou tractament.

FÀRMACS QUE ALTEREN EL CICLE DE LA SON:

Hi ha fàrmacs que alteren el cicle de la son, que, en cas de patir insomni es recomana l'administració d'aquets fàrmacs al matí en comptes de la tarda/nit.

FÀRMACS:

- DIÜRÈTICS
- VITAMINA B12
- IACE (inhibidors de l'Acetilcolinesterasa)
- Bupropion
- Vareniclina
- Fàrmacs psicoestimulants

Annexa 1. Tractament farmacològic per Clústers:

En el tractament dels SPCD generalment, ens podem trobar que un pacient no presenti un sol símptoma sinó que manifesti més d'un símptoma a la vegada. Per al tractament d'aquests símptomes es poden agrupar en clústers, permetent simplificar el tractament i evitant així l'ús excessiu de psicofàrmacs en el pacient amb demència.

Tipus de Clúster	Clúster 1	Clúster 2	Clúster 3
**Símptomes psicològics conductuals relacionats**	Irritabilitat<br>Ansietat/pànic<br>Humor depressiu	Agressivitat<br>Agitació psicomotorora<br>Psicosi	Inestabilitat afectiva<br>Impulsivitat<br>Agressivitat
**Tratamiento**	ISRS	Antipsicòtics	OXCARBAZEPINA

Annexa 2. Cohen – Mansfiel Agitation Inventori (CMAI)[26]

Sense agressivitat física	Amb agressivitat verbal
Inquietud psicomotora<br>Comportaments repetitius<br>Deambulació erràtica<br>Amagar objectes<br>Agafar i tocar tot<br>Síndrome crepuscular<br>Tendència a marxar<br>Vestir-se i desvestir-se en llocs inapropiats	Cridar<br>Maleir<br>Exabruptes<br>Comentaris socialment inapropiats
**Amb agressivitat física**	**Sense agressivitat verbal**
Colpejar<br>Empènyer<br>Esgarrapar<br>Agafar-se<br>Donar patades<br>Mossegar<br>Escopir	Negativisme<br>Canturejar<br>Frases repetitives<br>Interrupcions constats<br>Cridar l'atenció Constantment

Annexa 3. Taula relació de dosi recomanades i efectes adversos més rellevants fàrmacs antipsicòtics i antidepressius[27-37]

Antipsicòtic	Dosi diària	Sedació	SEP	ACH	Hipotensió Ortostàtica	DM
**RISPERIDONA**	0.5 – 2 mg	Baixa/ Moderada	Baix	Molt baix	Moderat	Baix/ Moderat
**QUETIAPINA**	12.5 – 100 mg	Moderada/ alta	Molt baix	Moderat	Moderat	Baix/ Moderat

Antipsicòtic	DREE	PROL	DT	LTR	Augment de pes	Lípids	QTc
**RISPERIDONA**	Si	Si	POC COMÚ	Baixa	Baix/ Moderat	Baix	Baix
**QUETIAPINA**	No	No	POC COMÚ	Baixa/ Moderada	Moderat	Moderat	Moderat

SEP: Efectes secundaris extrapiramidals
DM: Diabetis Mellitus
DREE: Efectes extrapiramidals dosi depenent
PROL: Elevació de la prolactina
DT: Discinèsia tardana
ACH: Efectes adversos anticolinèrgics
LTR: Increment dels resultats del test de funció hepàtica
Lípids: Elevacions del colesterol i/o triglicèrids
QTc: Prolongació interval QTc

Antidepressiu	Dosi inicial	Dosi mant	ACH	Somno-lència	Hipotensió Ortostàtica	GI	Augment de pes
**CITALOPRAM**	10mg matí	10-20 mg	0	0	0	3+	1+
**SERTRALINA**	12.5-50mg matí	50-100 mg	0	0	0	3+	1+
**TRAZODONA**	25mg/24h	100-150 mg	0	4+	3+	1+	2+
**MIRTAZAPINA**	7.5 mg nit	15-30 mg	1+	3+	1+	0	3+
**VENLAFAXINA**	25mg/24, 12 o 8h 37.5mg/dia(1)	75-150 mg 75(1)	1+	1+	0	3+	0

Puntuació: 0 efecte absent o estrany i 4+ efecte relativament comú
ACH: Efectes adversos anticolinèrgics
GI: Problemes gastrointestinals.
(1): forma farmacèutica de alliberació perllongada

Altres	Dosi inicial	Mareig/ vertigen	Somno lència	Atàxia	Fatiga	Hipona trèmia	GI
**OXCARBAZEPINA**	300/12-24h	22 – 49% 6 – 15%	20 – 36%	5 – 31%	12 – 15%	1 – 10%	7 – 36%
**CLOMETIAZOL**[36]	192 – 384 mg/nit	Irritació nasal, irritació conjuntival a vegades associat a mal de cap, increment de les secrecions bronquials o nasofaríngies, nàusees i vòmits, taquicàrdia i hipotensió. Dosis excessives pot produir: Coma, depressió respiratòria,					

			hipotensió, hipotèrmia, pneumònia seguida increment secreció respiratòria.		
**LORAZEPAM**	0.5-2mg /dia	1 – 10%	>10% (sedació)	1 – 10%	

Annexa 4. Taula maneig IACE[38-41]

IACE	Presentació	Titulació	Dosi Manteniment	Observacions
**DONEPEZILO**	Comp<br>Comp flash	5 mg/24h (4 setmanes)	10 mg/24h	Administrats **NIT abans de dormir, en cas d'insomni administrar al matí**
**GALANTAMINA**	Comprimits Lliberació perllongada	8 mg/24h (4 setmanes)<br>16 mg/24h (dosi manteniment)	16 – 24mg/24h	Administrats al **MATÍ AMB ALIMENTS**
	Solució oral (4mg/ml)	4mg/12h (4 setmanes)<br>8mg/12h (dosi manteniment)	8 - 12 mg/12h	Administrats **AMB ALIMENTS a l'esmorzar i al sopar**
	**IH MODERADA**	8mg/24h dies alterns (7 dies) administrar al **MATÍ**<br>8mg/24h (4 setmanes)	16mg/24h	Administrats al **MATÍ AMB ALIMENTS**
**RIVASTIGMINA**	Solució oral (2mg/ml)<br>Càpsules	1.5mg/12h (15 dies)<br>3mg/12h	3 – 6mg/12h	Administrats **AMB ALIMENTS a l'esmorzar i al sopar**
	Pegats	4.6mg/24h (4 setmanes)<br>9.5mg/24h	9.5mg/24h	Aplicació del pegat en la pell seca, neta intacte i sense pèl. En la zona del superior del braç, esquena o tòrax.
**MEMANTINA**	Comprimits	1a setmana 5mg/24h<br>2a setmana 10mg/24h<br>3a setmana 15mg/24h<br>4a setmana 20mg/24h	20 mg/24h	Administrats **sempre a la mateixa hora del dia cada dia**
	Solució oral (5mg/pulsació)	1a setmana 5mg/24h<br>2a setmana 10mg/24h<br>3a setmana 15mg/24h<br>4a setmana 20mg/24h	20mg/24h	La pulsació s'ha de posar en una cullera o en un got **MAI** directament a la boca. S'ha d'administrar **sempre a la mateixa hora del dia cada dia**

SEGUIMENT

- Revaluar al pacient en un període de temps màxim de 3 a 6 mesos.

- Si existeixen problemes de tolerància o falta d'efectivitat pot canviar-se de fàrmac o combinar-se els IACE con memantina.

- Es considera que un fàrmac és eficaç quan s'evidencia l'estabilització de la milloria dels símptomes que s'hagin escollit com a diana (cognitius, conductuals o funcionals).

EFECTES ADVERSOS

IACE	Somno-lència	HTA	Vertigen /mareig	Dispnea	Mal de cap	SEP	GI
**DONEPEZILO**	-	-	1 – 10%	-	≥ 10%	-	1 – 10%(1)
	<u>Altres RAM</u>: **Freqüents (1 – 10%)**: Síncope, Insomni, anorèxia, refredat comú, fatiga, dolor, Incontinència urinària, erupció, pruït, rampes musculars, al·lucinacions, agitació i conducta agressiva (es resol disminuint la dosi o retirant el medicament).						
**GALANTAMINA**	1 – 10%	1 – 10%	1 – 10%	-	1 – 10%	-	1 – 10%(2)
	<u>Altres RAM</u>: **Freqüents (1 – 10%)**: Síncope, anorèxia, fatiga, astènia, malestar, espasmes musculars, al·lucinacions, depressió, caigudes, bradicàrdia, hiperhidrosis, pèrdua de pes, tremolor i letàrgia.						
**RIVASTIGMINA**	1 – 10%	-	≥ 10%	-	1 – 10%	1 – 10%(3)	1 – 10%(4)
	<u>Pacients amb PARKINSON</u>: **Molt freqüents (> o = 10%):** Tremolor. **Freqüents (1 – 10%)**: Rigidesa muscular i trastorn de la marxa, Hipersecreció salivar, Insomni, intranquil·litat, ansietat, deshidratació, bradicàrdia.						
	<u>Altres RAM</u>: **Freqüents (1 – 10%):** Anorèxia, hiperhidrosi, agitació, confusió, fatiga, astènia, mal estar, pèrdua de pes.						
**MEMANTINA**	1 – 10%	1 – 10%	1 – 10%	1 – 10%	1 – 10%	-	1 – 10%(5)

GI: gastrointestinals

(1): **Molt Freqüents (> o = 10%):** Nàusees, diarrea. **Freqüents (1 – 10%):** Dolor abdominal, vòmits.

(2): **Molt Freqüents (> o = 10%):** Nàusees, vòmits. **Freqüents (1 – 10%):** Dolor abdominal, dispèpsia, mal estar abdominal i estomacal, diarrea.

(3): Més freqüents en pacients amb Parkinson: Empitjorament de la malaltia de Parkinson, Bradicinèsia, Discinèsia.

(4): **Molt Freqüents (> o = 10%):** Nàusees (38%), vòmits (23%), diarrea. **Freqüents (1 – 10%):** Dolor abdominal, dispèpsia.

(5): Estrenyiment

Annexa 5. Ajust de dosi en Insuficiència renal (IR) o Insuficiència hepàtica (IH)[27-41].

ANTIDEPRESSIUS	IR			IH
	Cl=5-29ml/min	Cl=30-49ml/min	Cl=50-80ml/min	
**CITALOPRAM**	< 20 ml/min Utilitzar amb precaució	No ajust de dosi	No ajust de dosi	Dosi Inicial 10mg/dia (dosi màxima 20mg/dia)
**SERTRALINA**	-	-	-	Pacients amb IH cas reduir la dosi o administrar-lo a intervals menys freqüents
**TRAZODONA**	PRECAUCIÓ	-	-	Precaució HEPATOTOXICITAT, monitorització funció hepàtica
**MIRTAZAPINA**	Cl <10ml/min Disminució clearance d'un 50% Ajustar la dosi	Cl=11-39ml/min Disminució clearance d'un 30%	-	Ajustament de la dosi, disminució clearance un 30%
**VENLAFAXINA**	Reduir 25 a 50% la dosi amb Cl = 10 – 70ml/min			IH moderada – severa, reduir la dosi 50%, en alguns pacients cal una reducció més elevada de la dosi.

ANTIPSICÒTICS	IR			IH
	Cl=5-29ml/min	Cl=30-49ml/min	Cl=50-80ml/min	
**RISPERIDONA**	Reduir a la meitat la dosi en cas d'IR moderada o severa		-	Reduir a la meitat de la dosi en cas d'IH
	L'ajust de la dosi en cas d'IR i/o IH s'ha de realitzar més lentament i amb precaució			
**QUETIAPINA**	-	-	-	Cal ajust de la dosi en cas d'IH
	L'ajust de la dosi ha de ser progressiva i molt lenta. L'aclarament de la Quetiapina està reduït d'un 30 a un 50% més respecte als pacients joves.			

IACE	IR			IH
	Cl=5-29ml/min	Cl=30-49ml/min	Cl=50-80ml/min	
**DONEPEZILO**	?	No ajust dosi	No ajust dosi	No recomanat en IH greu
**GALANTAMINA**	< 9ml/min CONTRAINDICAT	No ajust dosi	No ajust dosi	IH greu CONTRAINDICAT

**RIVASTIGMINA**	Monitorització ↑ les RAM	No ajust dosi	No ajust dosi	No recomanat en IH greu
**MEMANTINA**	10mg/dia	10mg/dia i si tolera provar 20mg/dia	No ajust dosi	No recomanat en IH greu

**ALTRES**	**IR**			**IH**
	Cl=5-29ml/min	Cl=30-49ml/min	Cl=50-80ml/min	
**OXCARBAZEPINA**	Cl < 30ml/min, reducció de la dosi inicial a la meitat.	-	-	No hi ha estudis en IH servera
**CLOMETIAZOL**	Utilitzar amb precaució			Utilitzar amb precaució
**LORAZEPAM**	No ajust dosi, però precaució	No ajust dosi, però precaució	No ajust dosi, però precaució	No ajust dosi, però precaució

BIBLIOGRAFIA

1. Scottish Intercollegiate Guidelines Network. Management of patients with dementia. A national clinical guideline. February 2006.

2. Steinberg M et al. Point and 5-year period prevalence of neuropsychiatric symptoms in dementia: the Cache County Study. Int J Geriatr Psychiatry 2008. 23; 170-177.

3. García-Alberca JM, Lara Muñoz P, Berthier Torres M. Neuropsychiatric and behavioral symptomatology in Alzheimer disease. Actas Esp Psiquiatr. 2010. 38(4): 212 – 222.

4. McShane R, Gedling K, Keene J et al. Getting lost in dementia: a longitudinal study of a behavioral symptom. Int Psychogeriatr 1998. 10(3): 253-60.

5. Gómez-Pavón J, González García P, Francés Román I, et al. Recomendaciones en la prevención de reacciones adversas a medicamentos en personas mayores con demencia. Rev Esp Geriatr Gerontol 2010. 45(2): 89-96.

6. Pelegrin Valero C. Marta Moreno E, Corbera Almajano M. Tratamiento sintomático de las demencias, Actas Esp Psiquiatr Monogr 2005. 3: 18-31.

7. Logsdon RG. McCurry SM, Teri L. Evidence-based psychological treatments for disruptive behaviors in individuals with dementia. Psychol and Aging 2007. 2: 28-36.

8. Teri L. Gibbons LE, McCurry SM, Logsdon RG, Buchner DM, Barlow WE et al. Exercise plus behavioral management in patients with Alzheimer's disease: a randomized controlled trial. JAMA 2003. 290: 2015-2022.

9. Gay KF. Managing agitation and difficult behavior in dementia. Clin Geriatr Med 2004. 20: 69-82.

10. Guía de práctica clínica sobre la atención integral a las personas con enfermedad de Alzheimer y otras demencias. Guías de práctica clínica en el SNS. Minsterio de sanidad, Politica Social e Igualdad. 2011.

11. Brodaty H, Burns K. Non-pharmacolgical Management of Apathy in Dementia: A systematic review. Am J geriatr Psychiatry 2011. 00: 1 – 16.

12. Berman K, Brodaty H, Withall A, Seeber K. Pharmacological treatment of Apathy in Dementia. Am J geriatr Psychiatry 2011. 00: 1 – 19.

13. Sink KM, Holden KF, Yaffe K. Pharmacological Treatment of Neuropsychiatric Symptoms of Dementia. A review of Evidence. JAMA 2005. 293(5): 596 – 608.

14. Zec RF, Burkett NR. Non-pharmacological and pharmacological treatment of the cognitive and behavioral symptoms of Alzheimer disease. NeuroRehabilitation 2008. 23: 425 – 438.

15. Omelan C. Approach to managing behavioral disturbances in dementia. Can Fam Physician 2006. 52: 191 – 199.

16. Ballard C, Howard R. Neuroleptic drugs in dementia: benefits and harm. Nat Rev Neurosci 2006. 7: 492-500.

17. Schneider LS, Dagerman K, Insel PS. Efficacy and adverse effects of atypical antipsychotics for dementia: meta-analysis of randomized, placebo-controlled trials. Am J Geriatr Psychiatry 2006. 14: 191-210.

18. La neuropsiquiatría de la enfermedad de Alzheimer y demencias relacionadas. Cummings JL. Atlas Medical Publishing Ltd. 2004. Edición española.

19. Teri L., Logsdon RG, McCurry SM. Nonpharmacologic treatment of behavioral disturbance in dementia. Med Clin of North Am 2002. 86: 641-656.

20. Banerjee S. The use of antipsychotic medication for people with dementia: time for action. A report for the Minister of State for Care Services. Department of Health, London, 2009.

21. Vitiello MV, Borson S. Sleep disturbances in patients with Alzeheimer's disease. CNS Drugs. 2001: 15(10): 777 – 796.

22. Zec F R, Burkett NR. Non-pharmacological and pharmacological treatment of the cognitive and behavioral symptoms of Alzheimer disease. Neuro Rehabilitation 23 (2008): 425 – 438.

23. E Delgado, M Muñoz, B Montero, C Sánchez, PF Gallagher, A Cruz-Jentoft. Prescripción inapropiada de medicamentos en los pacientes mayores: los criterios STOPP/START. Rev Esp Geriatr Gerontol. 2009; 44(5): 273-279.

24. Jones RM, Arlidge J, Gillham R, Reagu S, Van den Bree M, Taylor P. Efficacy of mood stabilizers in the treatment of impulsive or repetitive aggression: systematic review and meta-analysis. Br J Psych. 2011. 198: 93 – 98.

25. Valle Fernandez J. Estabilizadores del estado de ánimo. En: de la Serna de Pedro I, ed Psicofármacos en geriatría. Barcelona: Ars Medica 2006.

26. Cohen-Mansfield J. Agitated behaviors in the elderly: II-Preliminary results in the cognitively deteriorated. J Am Geriatr Soc 1986. 34: 722-727.

27. TP Sembla, JL Beizer, MD Higbee. Geriatric Dosage Handbook. 17th ed. Lexicomp Drug Reference Handbooks. 2012.

28. Fitxa tècnica Risperdal [consultada abril 2012] http://www.aemps.gob.es/cima/especialidad.do?metodo=verFichaWordPdf&codigo=65696&formato=pdf&formulario=FICHAS&file=ficha.pdf

29. Fitxa tècnica de Quetiapina Alter EFG [Consultada abril 2012] http://www.aemps.gob.es/cima/especialidad.do?metodo=verFichaWordPdf&codigo=69556&formato=pdf&formulario=FICHAS&file=ficha.pdf

30. Fitxa tècnica Citalopram Stada EFG [Consultada abril 2012] http://www.aemps.gob.es/cima/especialidad.do?metodo=verFichaWordPdf&codigo=66035&formato=pdf&formulario=FICHAS&file=ficha.pdf

31. Fitxa tècnica Sertralina Edigen EFG [Consultada abril 2012] http://www.aemps.gob.es/cima/especialidad.do?metodo=verFichaWordPdf&codigo=66049&formato=pdf&formulario=FICHAS&file=ficha.pdf

32. Fitxa tècnica Deprax [Consultada abril 2012] http://www.aemps.gob.es/cima/especialidad.do?metodo=verFichaWordPdf&codigo=51612&formato=pdf&formulario=FICHAS&file=ficha.pdf

33. Fitxa tècnica Mirtazapina Mylan EFG [Consultada abril 2012] http://www.aemps.gob.es/cima/especialidad.do?metodo=verFichaWordPdf&codigo=69077&formato=pdf&formulario=FICHAS&file=ficha.pdf

34. Fitxa tècnica Venlafaxina Bexal EFG [Consultada abril 2012] http://www.aemps.gob.es/cima/especialidad.do?metodo=verFichaWordPdf&codigo=66702&formato=pdf&formulario=FICHAS&file=ficha.pdf

35. Fitxa tècnica Oxcarbazepina Normon EFG [Consultada abril 2012] http://www.aemps.gob.es/cima/especialidad.do?metodo=verFichaWordPdf&codigo=73651&formato=pdf&formulario=FICHAS&file=ficha.pdf

36. Martindale. The Extra Pharmacopoeia. 31th edition.

37. Fitxa tècnica Lorazepam Cinfa EFG [Consultada abril 2012] http://www.aemps.gob.es/cima/especialidad.do?metodo=verFichaWordPdf&codigo=68477&formato=pdf&formulario=FICHAS&file=ficha.pdf

38. Fitxa tècnica Aricept [Consultada abril 2012] http://www.aemps.gob.es/cima/especialidad.do?metodo=verFichaWordPdf&codigo=68214&formato=pdf&formulario=FICHAS&file=ficha.pdf

39. Fitza tècnica Reminyl [Consultada abril 2012] http://www.aemps.gob.es/cima/especialidad.do?metodo=verFichaWordPdf&codigo=66652&formato=pdf&formulario=FICHAS&file=ficha.pdf

40. Fitxa tècnica Exelon [Consultada abril 2012] http://www.ema.europa.eu/ema/index.jsp?curl=pages/medicines/human/medicines/000169/human_med_000777.jsp&mid=WC0b01ac058001d124&jsenabled=true

41. Fitxa tècnica Axura [Consultada abril 2012] http://www.ema.europa.eu/ema/index.jsp?curl=pages/medicines/human/medicines/000378/human_med_000665.jsp&mid=WC0b01ac058001d124
