# Supplementary material for: Consensus and evidence-based medication review to optimize and potentially reduce psychotropic drug prescription in institutionalized dementia patients
Source: BMC Geriatr. 2019 Jan 8;19:7. doi: 10.1186/s12877-018-1015-9 (PMC6323667; doi:10.1186/s12877-018-1015-9)
Supplement: Supplementary file 2 — Brief summary of the consensus guidelines and development of the pharmacological review intervention for the management of BPSD. (PDF 46 kb) [file 12877_2018_1015_MOESM2_ESM.pdf]

Brief summary of the consensus guidelines and development of the pharmacological review intervention for the management of behavioral and psychological symptoms related to dementia (BPSD)

Considerations for pharmacological and non-pharmacological management of BPSD	Proposed action
**1. Identification of the symptom to treat**	
Could the symptom be due to a drug-related adverse effect?	Evaluate suspension or substitution of the drug, assessing the benefit/risk balance
Is there a trigger for the behavior or some organic problem that may be causing it?	Resolve the trigger for the behavior or treat the underlying health problem
**2. Identification of the BPSD to treat**	
Is there a non-pharmacological management approach?	Apply a non-pharmacological approach to avoid exacerbation of the symptom
Is it a symptom that can respond to pharmacological management?	**General considerations when prescribing a psychotropic drug**<br>**1. NEED**: Is a psychotropic drug needed to treat this symptom?<br>**2. SIMPLIFYING THERAPY**: If the patient has 2 or more BPSD, can the patient be treated with a single psychotropic drug?<br>**3. LOW DOSE**: Use of the minimum effective dose<br>**4. SAFETY**: Is this psychotropic drug associated with adverse effects more often in older people?<br>**5. REVIEW OF THE MEDICATION:** Based on what criteria and how often will the effectiveness of the treatment be reviewed? Essential for those symptoms that may not respond to treatment.

3. Recommendations for pharmacological and non-pharmacological management

The recommendations were based on evidence contained in the related literature and were established by consensus between care levels. The guidelines contain information on the Stopp-Start criteria, and separate algorithms and information for non-pharmacological and pharmacological management of each of the following BPSD: apathy, anxiety, depression, insomnia, psychosis, aggression, and agitation.

In annexes to the document there is information on pharmacologic treatment of symptom clusters, the Cohen-Mansfield Agitation Inventory, and tables describing the recommended dose and most common adverse effects of antipsychotic and antidepressant drugs, dose adjustment for renal failure, and other relevant information.

Application of these guidelines in the medication review

1. Evaluate dementia patients prescribed ≥1 psychotropic drug for **≥3 months**

- Clinical assessment: determine whether the patient has comorbidities or is in an end-of life state
- Assessment of dependence: Barthel index
- Cognitive assessment: confirm the diagnosis of dementia: Pfeiffer Test and Global Dementia Scale

2. Medication review

a. **Indication**: For what reason was the psychotropic drug prescribed?
b. **Effectiveness:** Has this psychotropic drug been effective for controlling the symptom? Is this psychotropic drug recommended for treating this symptom?
c. **Safety:** Is there therapeutic duplication, a contraindication due to age or comorbidity, an interaction, a drug-related adverse effect, or a prescribing cascade?
d. **Appropriateness:** Is the dose and dosing interval appropriate for this patient?
e. **Resolution:**
    a) **If the medication is not effective:** It is recommended to withdraw the drug or substitute it for another, more appropriate option, following the guideline recommendations.
    b) **If there is a safety-related incident:** It is recommended to withdraw the drug or substitute it for a safer option

c) **If the medication requires adjustment:** It is recommended to adjust the dose, dosing interval, or duration of the psychotropic drug.

d) **If the patient is stable:** Can withdrawal of the psychotropic drug be evaluated?

e) **If the patient has 2 or more symptoms that have to be treated:** Can the patient be treated with a single psychotropic agent?

f) **If the patient was treated with an acetylcholinesterase inhibitors or/and memantine we considered for deprescription** when the state of the dementia patient was rated as GDS-FAST ≥7b, with Karnofsky score <30 and with 3 criteria for advanced chronic disease: albumin ≤25g/L, multiple comorbidities, recurrent fever or stage III-IV pressure ulcers.
